# Healthcare for individuals without health insurance in Germany – a mixed methods approach to assess the situation and current challenges

**DOI:** 10.1186/s12939-023-01930-6

**Published:** 2023-06-19

**Authors:** Mathilde Stötzler, Andrea Kaifie

**Affiliations:** Institute for Occupational, Social, and Environmental Medicine, Medical Faculty, RWTH Aachen University, Pauwelsstrasse 30, 52074 Aachen, Germany

**Keywords:** Medical care, Healthcare, Health insurance, Health inequality, Pregnancy, Uninsured migrants, Undocumented migrants, EU citizens, MediNetz, Anonymer Krankenschein

## Abstract

**Background:**

Health insurance is mandatory in Germany; nevertheless, many individuals there have no health insurance and depend on a parallel healthcare structure. Voluntary associations, such as MediNetz and healthcare vouchers (“Anonymer Krankenschein” - AKS), support uninsured citizens. This study aimed to provide insights into associations, such as MediNetz and AKS that provide healthcare for individuals without health insurance in North Rhine-Westphalia, the largest federal state in Germany.

**Methods:**

A mixed methods approach was chosen. A qualitative study using interviews with experts was performed to gain their knowledge and explore the various challenges that AKS and MediNetz associations faced and the possible improvements that could be made. A quantitative survey was conducted to analyse the demographic data of the patients who required AKS or MediNetz’s assistance and the characteristics of each association through a separate questionnaire. Data was received from the association in Aachen, Bielefeld, Bonn, Düsseldorf, and Essen.

**Results:**

More women than men sought medical care; most were between 25 and 49 years old. The proportion of individuals without residency status accounted for the largest share (53.6%). Common reasons for patients to make contact were acute illnesses (40.2%) and pregnancies (22.3%). Most patients were sent to gynaecologists and general practitioners. Asking the experts, it became apparent that the existing system could not reach the standard of the regular healthcare in Germany. Financial and human resources were insufficient. Therefore, prevention was limited, especially chronically ill patients or patients with a severe illness requiring hospitalisation could not be treated. AKS had advantages compared to MediNetz, as the care came closer to the expected German medical standards.

**Conclusions:**

The results showed a demand for associations providing healthcare for individuals without health insurance. However, the healthcare provided by MediNetz and AKS did not reach the standard of healthcare in Germany and mainly depended on the organisations’ resources. Funded projects such as an AKS combined with clearing centres significantly improved healthcare. Until structural measures are implemented, they can be a transitional solution by spreading nationwide.

**Supplementary Information:**

The online version contains supplementary material available at 10.1186/s12939-023-01930-6.

## Background

The Universal Declaration of Human Rights states that “Everyone has the right to a standard of living adequate for the health and well-being of himself and his family, including food, clothing, housing and medical care […]” [1]. Moreover, the United Nations Committee on Economic, Social and Cultural Rights (CESCR) emphasised that states are obliged to provide those who do not have sufficient means with the necessary health insurance to prevent any discrimination in providing healthcare and health services [2].

Having health insurance is mandatory in Germany; nevertheless, many individuals there have no health insurance and thus have limited or no access to healthcare. In 2019, according to a report by the German Federal Statistics Office based on a micro-census (“small population census”) data, around 61,000 inhabitants were living without health insurance. The report also showed that self-employed and unemployed persons were particularly affected, with 0.4% of all self-employed and 0.8% of all unemployed individuals impacted [3]. The number of unreported cases is estimated to be significantly higher, as the report fails to take into account undocumented migrants in Germany.

In Germany, many individuals without access to healthcare are undocumented migrants (UMs) [4, 5]. There are several reasons why a migrant can be undocumented: (1) when a migrant illegally enters a country, (2) when a migrant arrives with a valid residency title, such as a tourist visa or a residency permit, but does not leave the country after the expiration date, (3) when the asylum application is rejected, and the applicant does not leave the country or (4) when an individual does not have a birth certificate, for example, when undocumented parents avoid applying for one out of fear of deportation [6].

UMs are legally entitled to healthcare according to the Asylum Seekers Benefits Act, which includes treatment for acute illnesses and pain conditions, vaccinations, and prenatal care [7]. However, UMs risk deportation when claiming medical care, as the social welfare office shares the data with the Aliens Department. Also, the risk of being reported to the police after being treated for an emergency exists despite medical confidentiality [8]. Deportation can be temporarily suspended if individuals cannot leave the country for health reasons, such as a severe illness [9].

After Romania and Bulgaria joined the European Union (EU) in 2007, there was an increase in Germany of citizens without health insurance from the new EU countries in Germany [10, 11]. EU citizens can move freely in the EU and receive social benefits, including early diagnosis and treatment of illnesses and assistance during pregnancy and maternity. However, the social benefits have been restricted since January 2017. Unemployed EU citizens are only entitled to social benefits after five years of residency [12].

Additionally, individuals who cannot pay their health insurance due to insolvency or loss of income have a significantly restricted entitlement to benefits; only treatments for acute illnesses and pain conditions are reimbursed. In March 2022, this concerned almost 700,000 individuals. This also affected German citizens [13].

These groups have no access to regular healthcare. Hospitals must treat individuals without health insurance to avoid the criminal consequences of denying assistance during a medical emergency. The decisive factor is whether treatment must take place immediately [14]. However, the difference between a medical emergency and a postponable treatment is not always clearly definable [8]. Local public health agencies (LPHA) must offer or ensure anonymous counselling and examination of sexually transmitted diseases and tuberculosis [15]. The treatment of HIV/AIDS has to be guaranteed if the patient does not have the financial means. However, there is a gap between legislation and reality, as only a minority of LPHA provide treatment, and HIV/AIDS patients are often not treated for an extended period [16].

For everything else, the uninsured are dependent on a parallel healthcare structure. In Germany, voluntary associations, such as MediNetz, support uninsured citizens. MediNetz are non-governmental associations that provide anonymous treatment free of charge regardless of the patient’s residency status by cooperating with doctors having different specialities. Currently, MediNetz operates in 33 locations throughout Germany, primarily in middle-sized or large cities; and is predominantly staffed by volunteers and financed exclusively by donations (https://medibueros.org/)[17].

Bonn, Leipzig, and Thuringia currently have municipal or regional-funded test projects called healthcare vouchers (“Anonymer Krankenschein” - AKS) in cooperation with so-called clearing centres. The AKS is supposed to provide short-term access to healthcare in Germany for patients without health insurance. Clearing centres advise and support individuals to enable long-term access to healthcare, often securing coverage through health insurance or clarifying other cost coverage possibilities [18]. In addition, the medical contact points of Doctors of the World offer primary and specialised care, including paediatric, gynaecological, and psychiatric consultations, as well as consultations for chronically ill patients for individuals without health insurance and homeless patients. These programmes are available in Germany´s biggest cities, such as Berlin, Hamburg, and Munich[4].

The aim of this study was to provide insights into associations, such as MediNetz and AKS, that provide healthcare for individuals without health insurance in North Rhine-Westphalia, the largest federal state in Germany. Our main research questions were:

i. Who are the patients that seek healthcare via MediNetz and AKS in terms of sociodemographic characteristics (e.g. age, gender, residency status)?
ii. What are their medical conditions (acute vs. chronic medical conditions)?
iii. Is there a gap in the provision of healthcare for patients without health insurance?
iv. What are the main challenges for the associations that provide healthcare for uninsured patients?

## Methods

A mixed methods approach using an explanatory sequential design with quantitative and qualitative data was chosen. The qualitative data, in the form of semi-structured interviews, was merged within the quantitative dataset. At the stage of interpretation and reporting, a weaving approach for integrating through narrative was used.

### Data sources

**Fig. 1:**
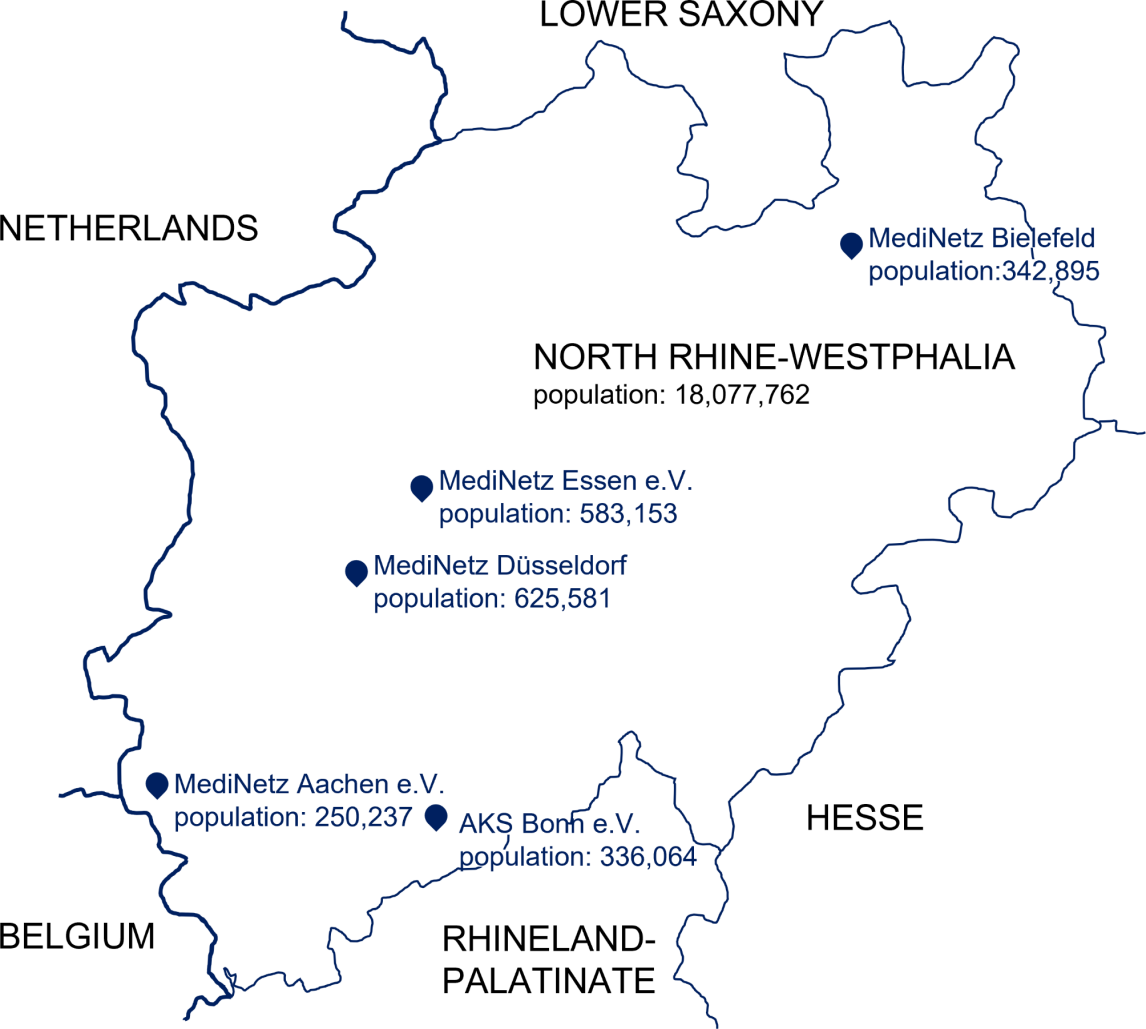
Map of North Rhine-Westphalia with the location of the cooperating associations and population data of the respecting cities from 30/06/22 [19, 20]

All MediNetz locations in North Rhine-Westphalia and AKS Bonn e.V. were contacted via email through their official contact channels in May 2022, and their collaboration was requested. This collaboration included an interview, the filling out of a questionnaire and the sharing of patients’ data. The response rate was five out of eight. Data was received from MediNetz Aachen e.V., MediNetz Bielefeld, AKS Bonn e.V., MediNetz Düsseldorf (STAY! Düsseldorfer Flüchtlingsinitiative), and MediNetz Essen e.V. (see Fig. 1).

### Patients’ characteristics (quantitative analysis)

All associations were asked to anonymously share their patients’ data regarding gender, age, country of origin, residency status, the specialists seen by the patients and the reasons for seeking medical care. The associations provided data for every year from 2016 to 2021, whereby the association in Düsseldorf had combined data from 2016 to 2018. The association in Bielefeld shared data from 2008 to 2021 for the specialists seen by the patients, and the association in Essen only shared data for 2021 due to missing documentation. AKS Bonn started its activity in November 2021, the data only ranged from November 2021 to August 2022. Data from n = 1519 patients was included in this analysis. All data was formatted into the smallest common denominator as there was no standardised data collection method among the different institutions.

The countries of origin were grouped into different regions based on the United Nations Geoscheme [21]. The age of the individual patients was available except for the ones from Bonn and Düsseldorf (for these associations, age was given in various categories). The data was grouped into the following categories: <18, 18–24, 25–49, 49–65, > 65, which the association in Düsseldorf used. The data from Bonn was converted accordingly. Residency status was summarised into the groups: no residency status, toleration or in the asylum procedure, tourist visa, EU citizen / German citizenship, missing and others.

The associations in Aachen and Bielefeld also shared individual patients’ data on reasons for seeking medical care. In Aachen, data was available from n = 195 patients from 01/2016 to 12/2021. In Bielefeld, data on n = 179 patients from 01/2017 to 04/2021 was obtained. The various diseases and symptoms were grouped into the following categories: acute complaints (infection, pain, wound), chronic illness / tumour, other / unknown, pregnancy, preventive care / vaccination, and psychological complaints. To compare our data with analyses of other providers in Germany, information regarding gender, age, and country of origin from open.med Berlin, westend open.med in Hamburg and open.med München of the years 2017 to 2021 was included in the results and discussion section using Doctors of the World reports [4, 22–25].

The data was analysed using SAS software (SAS 7.1, SAS Institute Inc., Cary, NC, USA) and Microsoft Excel (2023).

### Organisation´s characteristics (quantitative analysis)

A questionnaire was sent to all associations (n = 5) and contained 15 closed questions with either numeric values or multiple-choice answer options. It included questions regarding the following areas: (1) structure of the association, (2) finances with income and expenses, (3) members, (4) means of communication between patients and the association, and (5) clearing centres. There was also an option to add comments to most questions. The questionnaire was filled out by a member of the association. In all cases except Bielefeld, the person filling the questionnaire was the interviewee (see Table 1: I1, I3, I5, I6, I7). In Düsseldorf, the questionnaire was filled out by both employees and in Bielefeld by another member of the association.

**Table 1:** Characteristics of the experts who were interviewed

Association	Expert	Position of the expert	Professional background
AKS Bonn e.V.	I1	Employee	Student
MediNetz Aachen e.V.	I2	Member	Student
	I3	Member	Student
MediNetz Bielefeld	I4	Member	Student
MediNetz Düsseldorf	I5	Employee	Social worker
	I6	Employee	Social worker
MediNetz Essen e.V.	I7	Member	Student

### Expert interviews (qualitative analysis)

A semi-structured interview guideline was developed by both authors and piloted in advance with experts from the association in Aachen. The complete interview guideline can be found in the supplemental section. All interviews were carried out by one author (M.S.) who is also a member of MediNetz Aachen. All interviews were recorded and transcribed. It included the following categories: (1) patient characteristics (socio-demographic characteristics, follow-up, severity of illness), (2) cooperation with doctors and hospitals (procedure of finding new cooperations, different medical specialities), (3) difficulties (care of the patients, treatment of chronically ill patients, psychotherapy, screening) and (4) future/ suggestions for improvement. Most of the questions included sub-questions (see exemplarily Table 2).

**Table 2:** Example question of the interview guideline

Main question	Are there patients who cannot be treated or referred via MediNetz?
Subquestion 1	How are they characterised, e.g., by specific socio-demographic characteristics or certain diseases?
Subquestion 2	How do you deal with chronically ill patients with many follow-up visits?

Information on patient characteristics was sought to receive insights into the context regarding conspicuous data and to obtain additional information on the severity of medical conditions of some patients, which may have not been reflected in the dataset.

Six interviews were conducted with seven experts from five different associations from October 2022 to December 2022. Three experts were employees from state-funded institutions, and the remaining experts were volunteers (see Table 1). The expert from AKS Bonn e.V. was a member of MediNetz Bonn e.V. and was thus able to highlight the differences between the two associations. Two interviews were conducted in Aachen, as the first interview was too concise. In Düsseldorf, one interview was conducted with two experts simultaneously, both of whom work in the same association. The interviews were held and recorded via video using Zoom, except for both interviews in Aachen, which were recorded with a digital recorder in presence. The experts verbally gave their consent to the audio recording. Discussions ended when the expert had nothing more to add. After the interview, a post-interview protocol was written down for contextualisation. The interviews lasted, on average, 23 min (minimum: 16 min, maximum: 40 min).

The interviews were transcribed using speech-to-text software, with manual corrections. Afterwards, the respective answers were compared and analysed in an Excel spreadsheet through inductive category development. An example of the coding guideline can be found in the supplemental section. In the process, groups were summed together, and appropriate quotes were selected from both authors after discussion. The chosen quotes were then translated into English.

## Results

### Patients’ characteristics

**Fig. 2:**
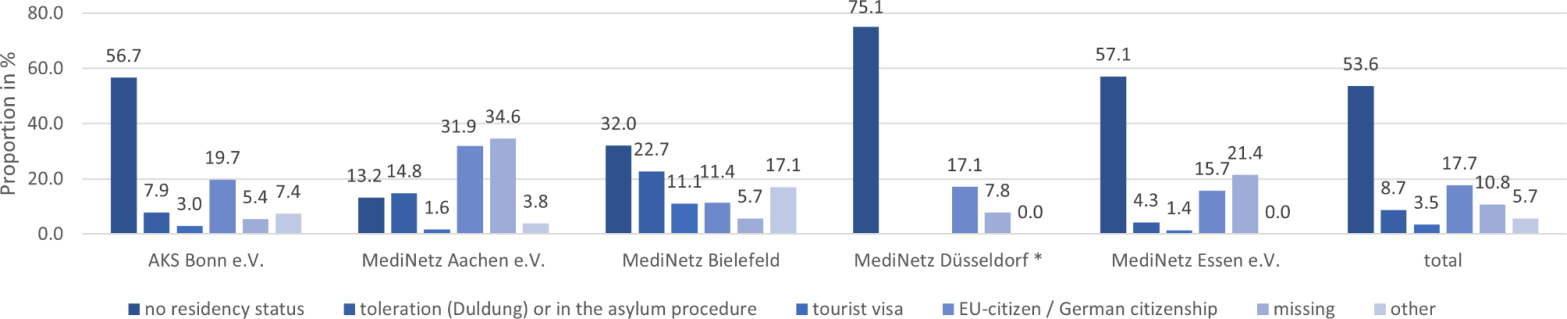
Residency status of the patients in each association in %; legend: * MediNetz Düsseldorf did not differentiate between types of legal migrants (toleration/ in the asylum procedure, tourist visa or EU citizen/ German citizenship)

The number of patients coming annually to each association varied depending on the city’s size and the association’s resources. The association in Aachen had the fewest, with about 45 patients per year (0.18‰ of Aachen’s population). In contrast, Bonn had the most, with an average of 250 patients per year (0.74‰ of Bonn’s population) (see Table 3).

**Table 3:** Characteristics of the patients using the association MediNetz, AKS and Doctors of the World (in Berlin, Hamburg and Munich); legend: * ^1^ age is known for 01/2016-05/2018; * ^2^ data was subsequently added after peer review * ^3^ age is known for the years 2017, 2019, and 2021; the age categories were indicated differently by Doctors of the World, instead of using the categories of < 18 and 18–24 years, the categories < 20 and 20–24 years were used; * ^4^ only the 7 most frequented countries were indicated in the years 2017–2019, 2021 [4, 22–25]

	AKS Bonn e.V.		MediNetz Aachen e.V.		MediNetz Bielefeld		MediNetz Essen e.V.		STAY Düsseldorf		total		Doctors of the World *²	
**time period**	11.2021–08.2022		2016–2021		2016–2021		2021		2016–2021				2017–2021	
**patients/ year**	∼250		∼45		∼60		∼100		∼170		∼125		∼2275	
**gender**	n = 203	%	n = 195	%	n = 179	%	n = 69	%	n = 671	%	n = 1317	%	n = 5386	%
female	125	61.6	129	66.2	112	62.6	38	54.3	423	63.0	827	62.8	2557	47.5
male	78	38.4	65	33.3	67	37.4	31	44.3	248	37.0	489	37.1	2820	52.4
diverse	0	0	1	0.5	0	0	0	0	0	0	1	0.1	9	0.2
**age**	n = 203		n = 195		n = 179		n = 70		n = 273*¹		n = 920		n = 3318*³	
< 18	12	5.9	12	6.2	16	8.9	4	5.7	17	6.2	61	6.6	764	23.0
18–24	30	14.8	22	11.3	34	19.0	13	18.6	28	10.3	127	13.8	255	7.7
25–49	80	39.4	72	36.9	79	44.1	28	40.0	170	62.3	429	46.6	1529	46.1
50–65	48	23.6	12	6.2	6	3.4	4	5.7	41	15.0	111	12.1	529	15.9
> 65	32	15.8	6	3.1	4	2.2	1	1.4	17	6.2	60	6.5	175	5.3
unknown	1	0.5	71	36.4	40	22.3	20	28.6	0	0.0	132	14.3	3	0.1
**region of origin**	n = 207		n = 195		n = 376		n = 70		n = 671		n = 1519		n = 5388*^4^	
Germany	17	8.2	7	3.6	2	0.5	0	0.0	0	0.0	26	1.7	691	12.8
Southern Europe	25	12.1	26	13.3	67	17.8	14	20.0	140	20.9	272	17.9	694	12.9
Eastern Europe	18	8.7	36	18.5	37	9.8	8	11.4	0	0.0	99	6.5	2132	39.6
North Africa	28	13.5	19	9.7	15	4.0	9	12.9	79	11.8	150	9.9	24	0.4
Remaining Africa	14	6.8	23	11.8	64	17.0	8	11.4	150	22.4	259	17.1	148	2.7
North/ Central Asia	0	0.0	0	0.0	5	1.3	0	0.0	0	0.0	5	0.3	0	0.0
West Asia	17	8.2	14	7.2	75	19.9	5	7.1	29	4.3	140	9.2	45	0.8
Remaining Asia	35	16.9	9	4.6	29	7.7	4	5.7	0	0.0	77	5.1	302	5.6
Central/ South America	45	21.7	2	1.0	2	0.5	0	0.0	11	1.6	60	3.9	0	0.0
others	8	3.9	8	4.1	1	0.3	1	1.4	0	0.0	18	1.2	1283	23.8
unknown	0	0.0	51	26.2	79	21.0	21	30.0	262	39.0	413	27.2	69	1.3
**most represented country**	Philipines: 26	12.5	Romania: 16	8.2	Albania / Kosovo: 31	8.2	Serbia: 7	10.0	Ghana:99	14.8			Bulgarien: 1368	25.6

#### Residency status

The proportion of individuals without residency status differed depending on the city and accounted for the largest share in all associations (53.6%) except for the one in Aachen. The second most significant proportion were EU citizens. The association in Aachen was the only one with more EU citizens (31.9%) than individuals without residency status (13.2%) (see Fig. 2). Due to city regulations, the association in Düsseldorf only allowed the referral to medical specialities of patients without residency status.

In the association in Aachen, a shift was seen over the past few years from UMs to citizens coming from the EU, mainly from Eastern Europe.

*“In the beginning, [...] we had many patients who were illegal and came from outside Europe, especially Syria and the Middle East. Now, we have more patients from Eastern Europe with health insurance problems but who are principally allowed to stay legally. [...] I think that this is on the one hand due to our cooperation. We cooperate, for example, with an association that supports individuals who work in prostitution. These are women, especially from Eastern Europe. On the other hand, perhaps because many of those who came to Germany with these so-called big waves are now in regular residency.”* (I2)

#### Gender

In all associations, more women than men sought medical care. Overall, the proportion of women was 62.8% (see Table 3).

#### Country of origin

The origin varied greatly between the associations. In Aachen, Romania was strongly represented with 8.2% of patients (16 patients); in Bielefeld, Albania/Kosovo, with 8.2% (31 patients); in Bonn, the Philippines, with 12.5% (26 patients); in Düsseldorf Ghana, with 14.8% (99 patients); and in Essen, Serbia with 10.0% (7 patients)). According to interviewees, this was possibly due to the different city communities and the association’s cooperation.

#### Age

Bielefeld had the highest proportion of people under 18 years (8.9%), while Bonn had a significant proportion of patients over 50 years (39.4%). However, most patients in all associations were between 25 and 49 years old (see Table 3).

#### Reasons for seeking medical care and severity of the illness

**Fig. 3:**
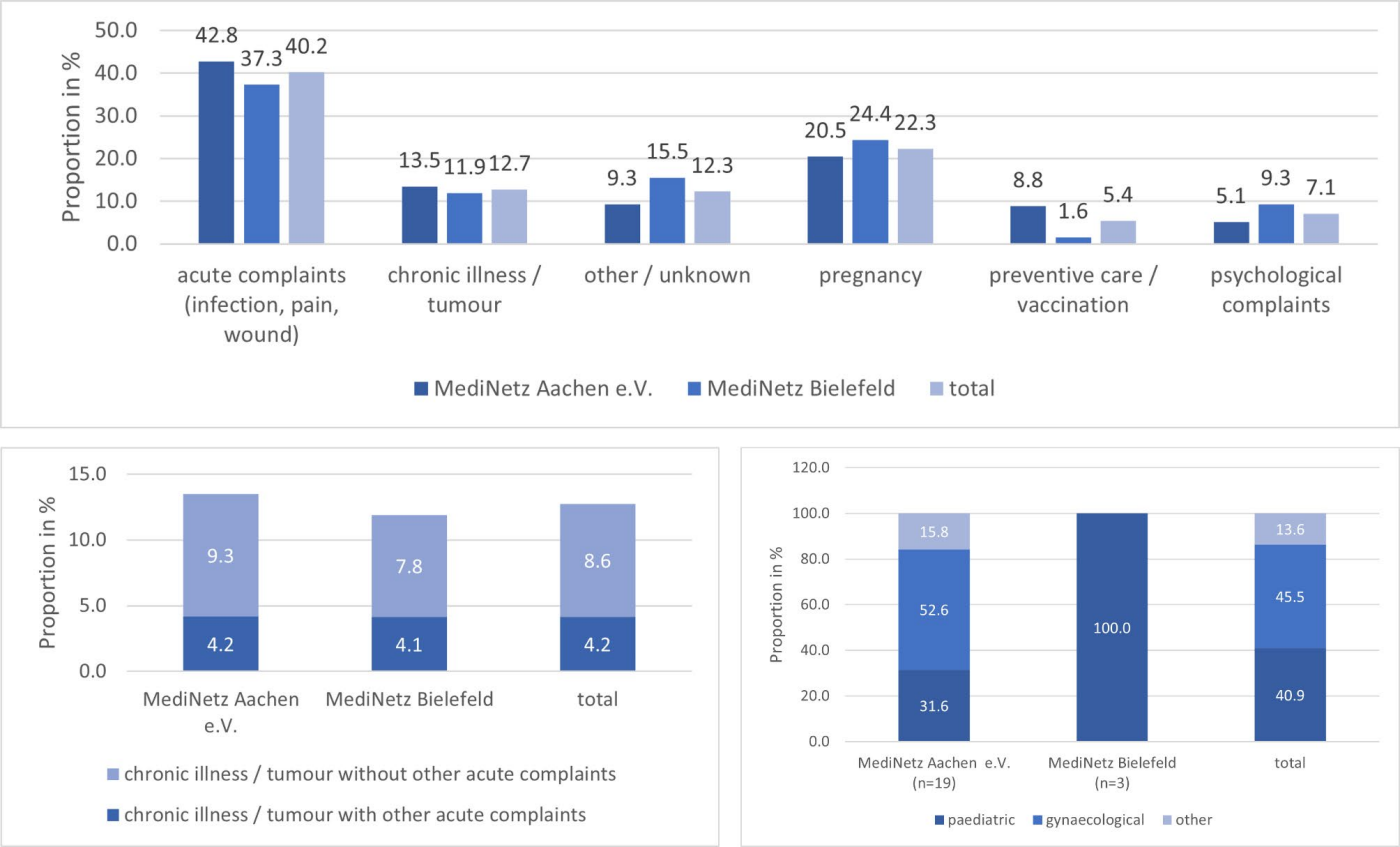
**a**: Reasons for seeking health care in the association in Aachen and Bielefeld in % (multiple reasons for one individual possible); **b**: preventive care / vaccination; **c**: chronic illness / tumour with or without other acute complaints

The reasons why patients contacted MediNetz were known for the associations in Aachen and Bielefeld. Most patients came due to an acute illness that could be treated through a few visits to a general practitioner. Of the n = 374 patients, only 8.6% used MediNetz due to chronic disease / tumour without other acute complaints. The most common reasons for patients to make contact were acute complaints (40.2%) and pregnancy (22.3%) (see Fig. 3). ~38% of all female patients in Bielefeld and Aachen were pregnant.

The severity of the illness varied; some patients came to MediNetz because of minor conditions and perceived the association as a general practice. However, some still waited long before asking for support, endangering themselves.

“*It varies. We have cases where people delay contacting us until the illness is severe, sometimes life-threatening or chronic. We also have cases where individuals died because they were afraid to seek medical help or because they were afraid of being deported. Now, however, we have patients who are well connected to us, and perceive us as a general practice in the broader sense and come to us with all their concerns.*” (I5)

Patients visited the AKS Bonn e.V. earlier than MediNetz Bonn e.V. Some came to use the clearing centre without being ill.

*“They usually only came to MediNetz when things were very bad, and they could not go on and had to see someone. That is more of a feeling than something we measure, but I would say that it has become more common for people to come to us earlier, sometimes even before something occurs or otherwise relatively early when it is not yet too bad*.” (I1)

### Cooperations with doctors and hospitals

**Fig. 4:**
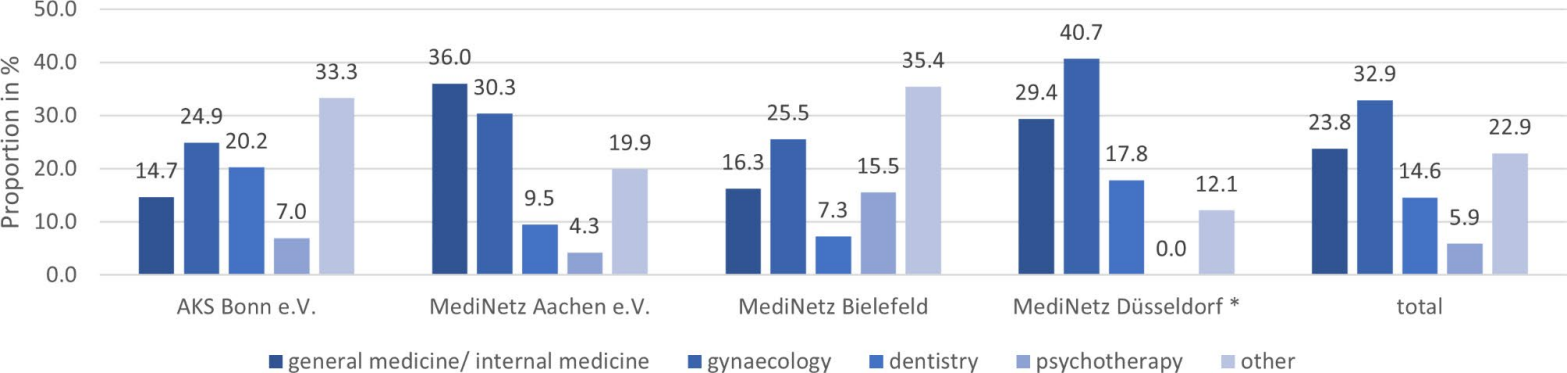
Cooperation with medical specialists in %; legend: * MediNetz Düsseldorf did not differentiate between “psychotherapy” and “other”

The cooperating doctors worked voluntarily, except for those collaborating with state-funded associations. In the association in Düsseldorf, the doctors were reimbursed at a lower rate than average. In the AKS Bonn e.V., the doctors were paid for the care they provided according to the pay rates. It was noticeable that all MediNetz had difficulties finding new cooperations with doctors. According to one interviewer, there was no speciality with enough doctors. The most common speciality to which the associations sent patients were gynaecology (32.9%), general medicine / internal medicine (23.8%), and dentistry (14.6%), except in the association in Bielefeld, where the third most common specialisation was psychotherapy with 15.5% (see Fig. 4). Much cooperation usually existed with general medical and gynaecological practices, but there was also a high demand for each. Due to pregnancies, many gynaecological doctors were needed, as the care is more intensive than for acute illnesses.

*“We have many cooperating gynaecologists; this year, however, we had 17 pregnancies that we have accompanied and are accompanying. […] It is different when we send someone to a dentist, who provides free treatment once than when a gynaecologist provides free treatment for the complete duration of the pregnancy. We see a problem, and a low-threshold contact point should be established.”* (I4)

The number and type of cooperation with hospitals varied greatly depending on the association. Two MediNetz cooperated with a hospital, taking over a certain number of births. The association in Essen had no such cooperation and could not finance births.

“*This is quite a frustrating aspect of our MediNetz work because we provide care for pregnant women but cannot afford birth. That means patients come to us, and we have to say on the first appointment that they will either go into debt or must try to get a health insurance. EU citizens sometimes find a solution through this pressure. It usually does not work out for citizens from Serbia and Northern Macedonia, who cannot get anywhere with an asylum application.”* (I7)

Many MediNetz cooperated with hospitals through which patients had no problem with registration because they were uninsured. Furthermore, they accepted MediNetz as the cost bearer, but the costs still had to be paid.

*“What I think is incredibly valuable for the patients [...] is that they are already registered beforehand. [...] That way, it goes smoothly and is less stressful for many patients. [...] From an administrative point of view, it is also valuable that we can sometimes ask beforehand how high the costs are and get answers because we have this limited static budget.”* (I6)

### Difficulties

It has become apparent that the existing system could not reach the standard of the regular healthcare in Germany.

“*Sometimes our resources are insufficient to treat the patients according to the guidelines, making two-class medicine necessary. We want to prevent that, but, unfortunately, that is impossible*.” (I3)

A reason for that was that there was no free choice of doctor. Furthermore, MediNetz were only accessible to a limited extent; patients could not be treated immediately in case of acute complaints.

#### Financial resources

In addition, the financial situation was a challenge. The association in Aachen, Bielefeld and Essen were purely financed by donations. Except for the association in Bielefeld, these associations did not have a clearing centre. The association in Bonn and Düsseldorf were state-funded and had salaried staff and a clearing centre attached to the association; the association in Bonn had more financial and human resources available. Nevertheless, all associations stated that their budgets were insufficient.

Budget constraints meant that many individuals, especially chronically ill patients or patients with a severe illness requiring hospitalisation, could not be treated.

*“In our case, the budget limit is challenging because we must keep weighing if a case is medically necessary, what we can pay for, and what is beyond our financial reach. For these reasons, we must assess each case ourselves, which we do not find reasonable. We want to have the greatest healthcare possible for people. We are not trained to make a medical assessment.”* (I6)

In the case of chronically ill patients, attempts were often made to cover part of the costs. For illnesses that did not cost as much, it could be covered. With more cost-intensive treatments such as hepatitis C or AIDS, this immediately became a problem.

*“This is a big problem with drugs. For example, we have several patients currently suffering from hepatitis C. In Germany, the medication costs 30,000 €, which does not fit our budget. The patients remain untreated, and severe liver diseases develop, often leading to death. (…) We cannot guarantee the supply of AIDS drugs continuously, which easily cost 1,000 € a month. We can pay it once a month or so to get things going. After that, it stops again, and they must get it through other donations.”* (I5)

#### Prevention and vaccination

Prevention was also limited. All MediNetz offered prenatal care and check-ups for children; other preventive care was partly provided. 5.4% of the patients in the association in Aachen and Bielefeld came to a screening/vaccination, apart from prenatal care. In Bielefeld, only 3 out of 179 patients went to a preventive check-up apart from prenatal care, all of which were paediatric. In Aachen, 19 out of 194 patients had a check-up apart from prenatal care; 52.6% were gynaecological, and 31.6% were paediatric (see Fig. 3). All patients in Aachen who had a gynaecological check-up came from Eastern Europe.

Vaccinations were partly covered, depending on the financial situation of the association. Vaccinations and other preventive care were covered in AKS Bonn as they paid for all treatments according to the catalogue of benefits of health insurance.

#### Municipal restrictions

The city’s restrictions posed a problem in state-funded associations because some individuals could not be treated.

“*It is particularly annoying that we can only treat some people. We must send many away. They often come to us because they have received the wrong information from another aid or a municipal organisation about what we can do. Then they come here, sometimes from other cities, and we must send them to another city.”* (I6)

#### Personnel resources and cooperation

The staffing situation was challenging for almost all associations except for those with employees. There were fluctuations in the personnel resources because the rest of the associations were purely voluntary, especially when the volunteers were students. The AKS had better accessibility and continuity because of full-time structures.

Moreover, one interviewee presented the increasing number of medical care units, so-called “Medizinische Versorgungszentren” (MVZ), as a problem, making it more challenging to find collaborations. Medical care units are characterised by an organisational separation of the ownership from the medical treatment activity, several doctors are employed. Their goal, achieved by facilitating interdisciplinary collaboration within a unified structure, is to enhance comprehensive patient care.

“*What is becoming more difficult for us is that there are more and more MVZs, medical care units. Our concept is that a freelance doctor is charitable and works with us. An MVZ is a business and will certainly have controllers. You must convince them; it is more difficult to convince companies than individuals to do such informal work, like in hospitals. That is something I am anxious about looking into the future.”* (I7)

Communication problems between patients and the association or doctors were also a problem because, among other things, of the language barrier.

#### Outlook on healthcare for uninsured individuals

The goal of all MediNetz was to dissolve themselves and to make the existing parallel structure superfluous, by making the government take responsibility instead. One option was to expand the concept of AKS to North Rhine-Westphalia or even the whole of Germany. Another possibility was finding a federal or state-level concept to provide similar and better care nationwide.

“*[…] that the system changes, so this parallel structure becomes superfluous. That would be the big goal, that it is also possible for people without a valid residency status to come, for example, into possession of an electronic health card and to have easy access to it. That could mean access to a national insurance number under a pseudonym. That would be the big vision.”* (I5)

Another suggestion for improvement was to have more flexible funding.

“*This static budget is complicated, as we keep experiencing. Ultimately, we are talking about a small group of patients coming to us […], not 10,000 or 100,000 or a million, but a three-digit number. Then, there are blatant statistical outliers because of the individual medical histories. Suddenly, […] there are three new patients with diabetes or AIDS or other things where continuous healthcare with medication quickly costs a few 1,000 €. To have a static budget of 100,000 € every year is very inconvenient.”* (I6)

Universal funding would enhance healthcare and personnel resources as well. Further suggestions for improvement were better communication structures and agreements with the patients and the doctors/hospitals, more personnel resources, and more public awareness of this topic.

“*I have to say that before I came into contact with MediNetz, I was unaware of how big an issue this is, of how many individuals do not have access to healthcare - although that is their human right.”* (I3)

## Discussion

To our best knowledge, this is the first study concerning the healthcare of patients without health insurance in Germany’s largest federal state. In conclusion, most of these individuals turn to MediNetz primarily due to acute health concerns or pregnancy. However, the ongoing treatment of chronic and severe illnesses requiring hospitalisation often remained uncertain.

### Patients’ characteristics compared to doctors of the World and other studies

Patients’ characteristics in the data from Doctors of the World differed from those in our dataset (Table 3). Most individuals were German citizens or EU citizens from other countries, primarily from Bulgaria and Romania [4, 22–25].

The available dataset showed a higher proportion of women, mainly due to pregnancy and delivery.

A higher proportion of women was also reported during a pilot project in Lower Saxony, where anonymous health insurance vouchers were issued. 55.9% of patients were women, almost half of them pregnant [26].

The proportion of male patients was found to be higher in Doctors of the World, possibly due to a significant number of homeless individuals seeking treatment (20.0% in 2021), who are predominantly male[25, 27].

Doctors of the World had on average a younger population compared to our dataset [4, 22–25]. Furthermore, there was an increase in the number of patients under five years old in the data of Doctors of the World. In 2017, 9.8% of patients were under the age of five, while in 2021 the number rose to 24.1%. Almost half of them were children of Vietnamese mothers and German fathers who faced considerable delays due to bureaucratic issues in Berlin [22, 25]. This may explain the high percentage of children under 18 years old in Doctors of the World.

Moreover, patients almost exclusively contacted an association because of acute complaints or pregnancy; only one out of ten people sought medical care because of a chronic illness without other acute complaints in the associations in Aachen or Bielefeld. One-third of those treated in the model project in Lower Saxony were found to have chronic illnesses, whose continued treatment was not secured due to the lack of a funding agency. The treatment of psychiatric disorders could not be guaranteed but was diagnosed in almost one-fifth of those treated [26]. However, it is important to treat chronic diseases to avoid relapse or worsening of an underlying chronic illness, such as diabetes mellitus or hypertension, which can have serious health implications if left undertreated.

When patients sought care, the severity of the diseases could be more advanced, and patients came on average later. Almost a fifth of initial consultations in Doctors of the World in the years 2017–2020 were assessed as requiring urgent or emergency care. Urgent cases refer to any condition that may escalate to an emergency if left untreated for more than 48 h [4, 22–24]. The degree of delay can be measured by the first visit for prenatal care. In the model project in Lower Saxony, the first visit was, on average, in the 17th week of pregnancy, and in medical contact points of “Doctors of the World”, only in the 20th week [4, 26]. At the same time, the first ultrasound examination is recommended between the 9th and 12th week of pregnancy [28]. The delay can lead to a late diagnosis and, thus, worse pregnancy outcomes.

### Difficulties and challenges of the associations

It was common practice in LPHAs (Local public health agencies) to refer cases with unresolved financial or legal challenges and medical problems to NGOs and physicians who provide pro bono or reduced-paid services [29]. However, voluntary associations could not provide care at an adequate standard. MediNetz services were only accessible to a limited extent. They depended on collaborations with doctors working pro bono, making a free choice of doctor impossible. Moreover, there was a big difference in what could be treated. In some associations that provided care, births, pre-natal and post-natal care could be covered entirely; in others, only part of the costs could be borne, and patients were sometimes forced to return to their home country or forced into debt. How much could be covered depended on the financial resources of the MediNetz, and the commitment of the cooperating doctors. Due to a limited financial budget, the associations were forced to decide who may be treated and who may not.

Even the AKS in Thuringia, which had a much higher budget than the voluntary associations, had to refuse treatments for cost reasons, such as inpatient stays [30].

Medical check-ups and vaccinations were offered to a limited extent; only paediatric check-ups and prenatal care were provided in all associations. Only a minority of patients came for medical check-ups in the associations in Aachen and Bielefeld. In addition to access to vaccinations and screening programs, health education and information for UMs were deemed insufficient [31].

Living without legal status harms health and well-being. Individuals with a more difficult life situation tend to have an increased risk of chronic diseases and a higher need for medical care [4]. The three main factors impacting health from a migrant perspective are: socio-economic conditions, the subjective experiences of criminalisation, and late presentation at healthcare facilities. Limited access to care may further exacerbate physical and mental illness [32].

The available dataset did not reveal any significant difference in the characteristics and number of patients during the COVID-19 pandemic, except for the closure of office hours in person in some associations, which made contact less personal and possibly more difficult for some patients. However, Doctors of the World reported that accessing healthcare was even more challenging for socially disadvantaged patients during the pandemic. Initially, UMs could not get tested or vaccinated as they lacked an identity card and an address. For chronically ill patients, who are susceptible to a severe COVID-19 disease, poor access to healthcare could be life-threatening. The COVID-19 pandemic has made healthcare inequalities more visible and has increased them. Early prevention and timely treatment could prevent emergencies and chronic diseases or at least mitigate their progression [4]. Moreover, treating a condition only when it becomes an emergency endangers a patient’s health and results in a more significant economic burden to the healthcare systems [33].

### Differences between AKS and MediNetz

According to this study, the AKS has advantages compared to MediNetz, as the care comes closer to the expected German medical standards. Vaccinations and preventive care were covered according to the catalogue of benefits of health insurance. The severity of diseases was less advanced compared to MediNetz. There was the free choice of doctor, and the doctors no longer worked pro bono. Because the financial situation was much better, practically no one had to be turned away due to a lack of financial resources. In addition, the number of individuals claiming the AKS was more significant compared to the size of the city than in the other associations, suggesting that the AKS had a much broader reach. According to the data of this study, the number of patients was still rising. Individuals in the association in Bonn were older than in the other associations, possibly because the care for chronic illnesses was better. Another advantage was that all patients were directly redirected to a clearing centre which tried to reintegrate patients into the health insurance system. However, the AKS Bonn e.V. could only treat patients from their city, as there was a requirement for patients to have lived in Bonn for at least three months before receiving treatment.

### Comparison with other countries

In other countries, there are different concepts concerning access to healthcare for uninsured individuals. A comparative study of national policies in 2012 grouped EU countries into three clusters based on the level of entitlement of UMs to healthcare. Cluster 1 included 10 EU member states, such as Finland and Ireland, which restricted entitlements to the extent that even emergency care was inaccessible for UMs, as it was considered not affordable. Cluster 2 included 12 EU member states in which UMs are entitled to minimum rights such as emergency care. Germany, Belgium, and Greece are in this category. Cluster 3 included 5 EU member states, such as France and Portugal, with more than minimum rights, where entitlements for UMs to healthcare services are beyond emergency care and include primary and secondary care [34]. The entitlements in these countries have been shifting. There was a growing tendency in Europe to restrict healthcare access for UMs and reinforce the link between access to health services and immigration control policies [35].

France, for example, has provided free healthcare through the State Medical Assistance (“Aide médicale de l’Etat”- AME) since 2000 for UMs living at least three months in France and being below a certain economic threshold [36]. At the end of 2018, 318,106 individuals benefited from the AME.

The demographic analysis of AME beneficiaries in 2018 showed that most were young, with 70.5% being strictly under 40 years and 54.3% being male. More than two-thirds of AME beneficiaries came from North and sub-Saharan Africa; Algerians were the most represented nationality. The medication differed from those of the French population, as the AME beneficiaries consumed more systemic antivirals (289%), anti-inflammatory and analgesic drugs, medications for addiction and drug dependence, anti-diabetics and antineoplastics [36].

A structural implementation analogous to AME is challenging because the organisation is mainly based on the work of the national health insurance fund and its branch in France. Since there are many statutory health insurance funds in Germany, the administrative processes would have to be taken over, for example, by the public health service or by newly established clearing centres. Healthcare financing would presumably have to continue to be organised via the social welfare office, provided anonymity or personal data protection would be assured [35].

Nevertheless, a nationwide solution should be found, as it is not the task of volunteers to solve the problem. Until structural measures are implemented, clearing centres with AKS with sufficient financial resources are a sensible temporary solution [4].

### Limitations

The data was collected from MediNetz and AKS. In Germany, several associations, such as Malteser Migranten Medizin or Caritas, provided care to individuals without health insurance. There were many unreported cases of individuals without health insurance since only sick patients used MediNetz. Furthermore, data was only collected from North Rhine-Westphalia; a different distribution may be seen throughout Germany and in larger cities. Since it was mainly women who used the MediNetz, men were underrepresented. In addition, we were not able to assess patient outcomes, such as morbidity or mortality after receiving healthcare to address differences between insured and uninsured individuals. Additionally, we acknowledge a potential conflict of interest given that a member of MediNetz conducted the interviews. We have consciously considered this while designing the study and especially while structuring the interviews to ensure the integrity and objectivity of the research findings.

## Conclusions

The results showed a demand for associations providing healthcare for individuals without health insurance. However, healthcare did not reach the expected standard in Germany and mainly depended on the organisations’ resources. Funded projects such as an AKS combined with clearing centres significantly improved healthcare. Until structural measures are implemented, they can be a transitional solution by spreading nationwide.

## Electronic supplementary material

Below is the link to the electronic supplementary material.

Supplementary Material 1

## Data Availability

Data can be made available upon request.

## List of abbreviations

AKS: Anonymer Krankenschein
AME: Aide médicale de l’Etat
EU: European Union
MVZ: Medizinische Versorgungszentren
NGO: Non-governmental organisation
UMs: undocumented migrants
