# Supplementary material for: Healthcare for individuals without health insurance in Germany – a mixed methods approach to assess the situation and current challenges
Source: Int J Equity Health. 2023 Jun 19;22:117. doi: 10.1186/s12939-023-01930-6 (PMC10280908; doi:10.1186/s12939-023-01930-6)
Supplement: Supplementary file 1 — Supplementary Material 1 [file 12939_2023_1930_MOESM1_ESM.docx]

**Suppl. 1: Expert Interviews**

Patients

- Which individuals make use of your MediNetz?
  - Is there a particular group of people that is prominently represented among the patients of your MediNetz?
  - What socio-demographic traits are exhibited by the patients who seek treatment at your MediNetz?
  - Are there any notable features related to the severity of illnesses among patients of your MediNetz?
- Is there a follow-up process for patients? If so, how is it done?

Cooperations

- What is the process for establishing cooperation with doctors?
- Which medical specialities are well-represented, and which are not, and why?
- Are there any cooperations with hospitals?

Difficulties

- What challenges do you encounter in the provision of care to patients through MediNetz? Please describe where these difficulties arise (patient characteristics, structural issues, communication barriers, etc.)
- Are there patients who cannot receive treatment through MediNetz?
  - How are these patients characterised, for example by certain sociodemographic characteristics or specific illnesses?
  - How are patients with chronic illnesses requiring frequent follow-up visits, patients with severe illnesses (e.g. tumours, a requirement of dialysis) or those needing psychotherapy managed? Could you share specific cases as examples?
- Are preventive screenings offered and utilised? If yes, which ones are available?

Outlook on healthcare for uninsured individuals

- Do you have any specific proposal for improving MediNetz?
- What do you wish for the future of MediNetz?

**Suppl. 2: Example of the coding guideline**

| **Category Cooperations** | | |
| --- | --- | --- |
| Subcategory 1 | Cooperation with doctors | |
| Additional rule | Doctors work voluntarily | Doctors are paid |
|  | *I would say that there is a need for more cooperation in most specialities. There are times when doctors are on vacation or someone is ill, especially during the Coronavirus pandemic. I can't think of any speciality where I would say we don't need more doctors.* | *The patient can visit any doctor of their chosen speciality.* |
| Subcategory 2 | Cooperation with hospitals | |
| Additional rule | One-time cooperation | Ongoing cooperation |
|  | *There has been cooperation for single cases, but they didn’t continue after. We once had a birth […], and the hospital cooperated. A few years later, we had another pregnant woman, and the same hospital didn’t cooperate.* | *What I think is incredibly valuable for the patients [...] is that they are already registered beforehand. [...] That way, it goes smoothly and is less stressful for many patients. [...] From an administrative point of view, it is also valuable that we can sometimes ask beforehand how high the costs are and get answers because we have this limited static budget* |
| Subcategory 3 | Cooperation in case of birth | |
| Additional rule | No cooperation | Cooperation exists |
|  | *This is quite a frustrating aspect of our MediNetz work because we provide care for pregnant women but cannot afford birth. That means patients come to us, and we have to say on the first appointment that they will either go into debt or must try to get a health insurance. EU citizens sometimes find a solution through this pressure. It usually does not work out for citizens from Serbia and Northern Macedonia, who cannot get anywhere with an asylum application* | *We cooperate with a smaller hospital, which has agreed to provide up to ten births per year, free of charge.* *For the hospital, the main concern is that many patients come emergently during childbirth, and it's very difficult to adequately support a woman during labour without any information, including whether a maternity record has been issued or if all the necessary examinations have been performed beforehand.* |
| **Category Difficulties** | | |
| Subcategory 1 | Financial resources | |
| Additional rule | No financial support, solely financed by donations | Financial support from the city |
|  | *Sometimes our resources are insufficient to treat the patients according to the guidelines, making two-class medicine necessary. We want to prevent that, but, unfortunately, that is impossible*.” | *In our case, the budget limit is challenging because we must keep weighing if a case is medically necessary, what we can pay for, and what is beyond our financial reach. For these reasons, we must assess each case ourselves, which we do not find reasonable. We want to have the greatest healthcare possible for people. We are not trained to make a medical assessment.* |
| Subcategory 2 | Prevention and vaccination | |
| Additional rule | Prevention and vaccination are partly provided | Prevention and vaccination are covered |
|  | *Vaccinations depend upon the circumstances of each case. Especially children require specialised vaccines which are expensive.* | *We pay for all treatments in alignment with the catalogue of benefits of health insurance.* |
| Subcategory 3 | Municipal restrictions | |
| Additional rule | No municipal restrictions | Municipal restrictions |
|  | *No, we do not refuse assistance to anyone. If we are unable to provide immediate aid, we ensure to guide individuals towards other suitable organisations. When someone reaches out to us, we never tell them we are unable to assist. We always try to arrange doctor appointments.* | *It is particularly annoying that we can only treat some people. We must send many away. They often come to us because they have received the wrong information from another aid or a municipal organisation about what we can do. Then they come here, sometimes from other cities, and we must send them to another city.* |
| Subcategory 4 | Personnel resources | |
| Additional rule | Volunteer based | Employee based |
|  | *Currently, our staffing situation is not particularly good. Due to the pandemic, we have gained fewer new members. Also, many have completed their studies and moved away.* | *The other aspect is that we have full-time structures. This is immensely beneficial for both accessibility and maintaining continuity.* |
| Subcategory 5 | Communication | |
|  | *Significant language barriers also pose a problem. When individuals cannot speak German, English, or French and do not have anyone to translate for them, we still offer telephone language mediation, but sometimes, that might not be sufficient.* | |
